# A Contribution to the Treatment of Versions and Flexions of the Unimpregnated Uterus

**Published:** 1871-12

**Authors:** 


*A Contribution to the Treatment of the Versions and Flexions of the Unimpregnated Uterus.* By Ephraim Cutter, A. M., M. D. Boston: James Campbell. 1871.

This pamphlet of 44 pages, contains a very able article on the above subject. It is illustrated by twenty wood cuts, showing the different abnormal positions of the uterus and the instruments and methods employed in its treatment. We recommend it to the general practitioner who will find it very interesting and instructive, containing full explanations of the most approved methods, of treating various uterine displacements.

